# Draft Genome Sequence of *Campylobacter ureolyticus* Strain CIT007, the First Whole-Genome Sequence of a Clinical Isolate

**DOI:** 10.1128/genomeA.00262-14

**Published:** 2014-04-10

**Authors:** Alan Lucid, Susan Bullman, Monika Koziel, Gerard D. Corcoran, Paul D. Cotter, Roy D. Sleator, Brigid Lucey

**Affiliations:** a Department of Biological Sciences, Cork Institute of Technology, Bishopstown, Cork, Ireland; b Department of Microbiology, Cork University Hospital, Wilton, Cork, Ireland; c Teagasc Food Research Centre, Moorepark, Fermoy, Cork, Ireland

## Abstract

Herein, we present the draft genome sequence of *Campylobacter ureolyticus*. Strain CIT007 was isolated from a stool sample from an elderly female presenting with diarrheal illness and end-stage chronic renal disease.

## GENOME ANNOUNCEMENT

*Campylobacter* is the most common cause of gastroenteritis worldwide, with *Campylobacter jejuni* and *Campylobacter coli* traditionally believed to be the main species associated with human illness. However, recent studies strongly suggest that *Campylobacter ureolyticus* may be a significant cause of gastroenteritis, being second only to *C. jejuni* as the most common cause of campylobacteriosis in southern Ireland (1, 2, 3). In the current study, we isolated *Campylobacter ureolyticus* strain CIT007 from the stool of an elderly female presenting with diarrheal illness and end-stage chronic renal disease at Cork University Hospital, Cork, Ireland.

*Campylobacter ureolyticus* strain CIT007 was sequenced using a combination of Illumina MiSeq with 250-bp paired-end reads and Roche 454 GS FLX+ single-end reads. Illumina sequencing generated 878,608 reads in pairs with an estimated genome coverage of approximately 200×, while the 454 platform yielded 104,653 reads, giving approximately 30× genome coverage. The reads were assembled *de novo* by means of a hybrid assembly approach using MIRA (4), resulting in 26 contigs. The total draft genome size is 1,665,887 bp and the estimated G+C content is 29.0 mol%. The genome was annotated using the annotation pipeline Prokka. A total of 1,677 coding sequences (CDS) were identified, with 403 assigned as hypothetical, accounting for 24% of all CDS. Of the total CDS, 116 encoded putative proteins predicted to be secreted using SignalP 4.1 (5).

Among the predicted CDS, several putative virulence factors were identified in accordance with our previously published work (6), including genes encoding putative efflux pumps involved in conferring antibiotic resistance (the multidrug and toxic compound extrusion [MATE] family multidrug efflux pump), the archetypal VirB/D4 type IV secretion system (TIVSS), adhesion-associated factors (fibronectin-fibrinogen binding protein), and invasion-associated factors (CiaB). Interestingly, while the TIVSS is most commonly associated with the pVir plasmid of *C. jejuni*, it appears to be chromosomally integrated in the *C. ureolyticus* genome. While these virulence factors are likely important drivers of gastrointestinal pathogenicity in humans (and other mammals), further studies are required to investigate the true pathogenic potential of this novel pathogen. Indeed, a current focus of our laboratory is the sequence analysis of multiple *C. ureolyticus* isolates from a variety of sources, both clinical and environmental, to determine how the pathogen grows and survives both inside and outside the host.

### Nucleotide sequence accession numbers.

This whole-genome shotgun project has been deposited at DDBJ/EMBL/GenBank under the accession number JFJK00000000. The version described in this paper is version JFJK01000000.
